# Working retirees in Taiwan: examining determinants of different working status after retirement

**DOI:** 10.1186/s12877-024-04849-x

**Published:** 2024-03-04

**Authors:** Tai-Kang Wu, Li-Jung Elizabeth Ku, Jer-Hao Chang, Ching-Ju Chiu, Susan C. Hu

**Affiliations:** 1 Department of Public Health, College of Medicine, National Cheng Kung University, No.1, University Road, Tainan, 70101 Taiwan; 2 Department of Occupational Therapy, College of Medicine, National Cheng Kung University, No.1, University Road, Tainan, 70101 Taiwan; 3 Institute of Gerontology, College of Medicine, National Cheng Kung University, No.1, University Road, Tainan, 70101 Taiwan

**Keywords:** Work after retirement, Retirees, Aging, Gender differences

## Abstract

This study aims to investigate the factors influencing the work status of retirees after retirement, especially focusing on self-employment and unpaid work. Data was taken and analyzed from the “Taiwan Health and Retirement Study,” a nationally representative sample of retired personnel aged 50–74 in 2015–2016. Four types of work status were classified after retirement: Fully retired, Paid work, Self-employment, and Unpaid work. Multinomial regression analysis was used to explore the factors related to participation in paid, self-employed, and unpaid work. Results show that pre-retirement occupation was significantly associated with paid work after retirement. For example, retirees in Taiwan who were employed by private enterprises or self-employed before retirement were more likely to engage in paid work after retirement than civil servants before retirement. Two other factors, namely pre-retirement job stress and work flexibility, prolong the careers of retired workers, especially in self-employment and unpaid work after retirement. Gender also significantly affects the choice of work after retirement. These findings can be used as a reference for future policies on the aging labor force.

## Introduction

According to the United Nations World Population Prospects Report [1], the global population continues to age rapidly, with the population aged 65 and over being the fastest-growing group, reaching 16% of the population by 2050.The process of aging is accompanied by an increase in life expectancy and a decrease in fertility rates, which may lead to a temporary increase in the proportion of the working-age population compared to the total population. Many countries have missed the window of opportunity for this demographic dividend, and the increasing elderly population poses challenges and pressures to various aspects of the national labor force, market economy development, fiscal revenue, elderly care, and pensions. According to the National Development Council of Taiwan [2], the working-age population accounts for 70.3% of the total population and is in the transitional stage of demographic transition. The island’s rate of aging is the highest in the world, and is about to enter a super-aging society by 2025. Therefore, it is necessary to immediately understand the labor force participation of the retired population and analyze the relevant factors that affect their continued work after retirement to facilitate the planning of the country’s future development.

Furthermore, the definition of retirement has gradually changed in modern society. Reaching 65 was often used as the physiological threshold for entering old age, and retirement was considered the end point of a person’s career as they exited the workforce. However, due to recent medical advancements, middle-aged and older adults experience a longer life expectancy and are more active after retirement. The previously-held definition of retirement is no longer applicable; instead, the decision to retire depends on the individual and can be viewed as a flexible transition process from work to completely leaving the labor market [3, 4] rather than a single, one-time event [5].

Recently, the proportion of elderly workers who continue to work after retirement has gradually increased. For example, more than half of workers in the United States choose to continue working after retirement [6]. Additionally, 15-26% of retired elderly workers return to the workforce [7, 8]. The modern social environment also provides greater flexibility and freedom in employment relationships [9]. Long-term contracts based on loyalty, security, lifelong employment, and mutual commitment have been replaced by ones based on short-term, low-loyalty, and more flexible work arrangements. Therefore, new flexibility, freedom, and autonomy have emerged during the later stages of workers’ careers.

Previous studies have often examined the transitions to different types of work after retirement, such as phased retirement, bridge employment, and re-entry after traditional retirement. For example, a cross-country study [10] divided the retirement process into complete retirement, the continuity type of post-retirement, and the hopping type. Complete retirement describes those without economic activity for living throughout the period and relying on pensions. The continuity type of post-retirement refers to a worker who resides in the labor market and relies on a public pension. The hopping type of post-retirement refers to an employee who repeatedly shifts between employment and non-employment in the labor market. However, these studies primarily focus on employed or paid work [11, 12] and often overlook the critical factors associated with unpaid work after retirement.

Unpaid work is another important social activity for retired individuals, providing productivity functions [13]. Norwegian scholars [14] pointed out that unpaid work is more closely related to a part-time job or full retirement than full-time paid work. These types of work may be complementary and dynamically adjusted. Research examining factors related to work after retirement has mainly focused on paid work, and our understanding of self-employment and unpaid work remains limited. Furthermore, most studies have been conducted in Western countries, which shows a continued lack of analysis on retired populations in Asia. Therefore, this study uses a dataset from the Health and Retirement Study in Taiwan to explore various antecedents and predictors of retirees engaging in different types of work after retirement to help provide critical information for active aging and social participation after retirement.

## The retirement systems in Taiwan

Taiwan is rapidly moving towards a super-aged society, with the post-war baby boomer population gradually retiring. According to statistics from Taiwan’s National Development Council, the country’s middle-aged and older adults have prematurely exited the labor market, resulting in a lower labor force participation rate for those over 55 years old than in OECD countries. Moreover, the proportion of Taiwan’s labor force aged 65 and above is much lower than that of major countries worldwide. For example, in 2021, the labor force participation rate of people over 65 in Taiwan was only 9.2%, compared with 18.9% in the United States and 36.3% in South Korea [15]. This difference may have a lot to do with Taiwan’s retirement system. The following is a brief introduction to Taiwan’s retirement system.

The retirement system in Taiwan is primarily composed of two tiers of occupation-based income security systems; one is for general workers, and the other is for civil servants, comprising pensions and elderly insurance benefits. For the pension system, most workers in the private sector are protected by the Labor Standards Act implemented in the early 1980s. This legislation includes retirement pension and eligibility requirements for voluntary and mandatory retirement (based on age and years of service). To qualify for voluntary retirement, workers need to meet one of the following conditions: (1) reaching 55 years old with 15 years of work, (2) completing 25 years of work, or (3) reaching 60 years old with 10 years of work.

In 2005, the Labor Retirement Pension System was newly instituted. The primary difference between the old and new Labor Retirement Pension systems lies in the transferability of workers’ years of service between employers, a restriction present in the old system but eliminated in the new one. The civil servants’ retirement pension system originated in the early 1940s. It underwent reform in 1995, transitioning from a system purely funded by the government to a retirement fund jointly supported by the government and employees (including civil servants, public school educators, and military personnel). Noticeably, the mandatory retirement age in both systems is set at 65 in Taiwan [16].

For the elderly insurance system, workers can apply for related benefits when participating in specific occupational insurance programs such as labor insurance, civil servants insurance, school staff insurance, and military personnel insurance. A common feature of these occupation-based insurance plans is that retirees must meet the age and work-year requirements stipulated in the regulations. Therefore, the eligibility criteria for elderly insurance benefits vary depending on the plan. Regarding working after retirement, there is no restriction on continuous work after receiving a pension in Taiwan. However, civil servants who want to continue working in public institutions are still subject to basic salary restrictions.

## Theoretical framework

Retirement is a comprehensive concept encompassing various subjective and objective states. It can be broadly categorized into five types: (1) defining retirement status based on individual subjective assessment; (2) categorizing retirees based on affirmative responses regarding their economic activity; (3) defining retirement based on the time retirees exited their primary work, i.e., the longest time spent working in that job; (4) defining retirement based on the time retirees began receiving public or corporate pensions; (5) defining retirement based on reduced working hours [10].

In this study, we adopted the fourth approach to define post-retirement work situations, especially examining the economic activity from the time of pension receipt until the present. However, constrained by the cross-sectional data format, we could not further classify the retirement process into the hopping type of post-retirement, instead categorizing them into four post-retirement work situations: paid work, self-employment, unpaid work, and complete retirement. The most common theoretical frameworks for exploring the antecedents of continued work after retirement are life course, role theories, and life cycle models.

## Life course theory

Life course theory emphasizes that life transitions do not occur independently but are shaped by the overall impact of social environments [17]. Therefore, life course theory can be used as a framework herein to clarify the antecedents of continuing work after retirement. The retirement process may be influenced by individual attributes and backgrounds such as gender, education, health status, financial status, and work experience before retirement. Existing models often include gender as one of the variables for analysis.

## Role theory

Role theory believes that role transitions can result in either positive or negative adjustment outcomes depending on whether they fit an individual’s values or goals [18, 19]. The theory emphasizes the influence of work-related psychological factors on retirement decisions, such as work stress, high job demands, relationships with colleagues/supervisors, and job autonomy. Role theory also acknowledges that gender roles may be part of one’s work identity, thereby affecting post-retirement behavior and the decision to continue working [20].

### Life cycle model

The lifecycle model focuses on individuals’ economic and financial decisions across different life stages. This model assumes that people’s behavior is affected by life stages, income, family circumstances, employment status, etc., and predicts individuals’ saving, investment, and spending behaviors at different stages of life. It examines how individuals cope with income changes, retirement planning, and consumption expenditures across different stages and typically employs economic models to assess these decisions [21].

The current study, which draws from Zhan, Wang, and Shi [22] and Beehr and Bennett [23], aims to examine factors influencing the decision to continue working after retirement at both individual and contextual levels. The individual level encompasses personal characteristics such as age, gender, education, marital status, and health. The contextual level comprises work-related factors such as pre-retirement occupation, work-related stress, flexibility, and autonomy. In accordance with the life cycle model, economic reserves influence retirement decisions; hence, financial status and the types of pension benefits received are also analyzed. Furthermore, given that gender may lead to different roles and life trajectories, this study will independently examine its impacts on the influencing factors and differences.

## Methods

### Dataset and participants

This study utilized the Taiwan Health and Retirement Study (THRS) dataset completed by the Center for Health Cities Research at National Cheng Kung University. The THRS was funded by the Health Promotion Administration of the Ministry of Health and Welfare in Taiwan to understand retirement planning and its effect on the health of retirees. The survey sample was nationally representative, and the respondents were retired civil servants and labor insurance personnel aged 50–74 from Taiwan (excluding those from outlying islands). The sample size was distributed according to the proportion of age distribution, geographical location, and urbanization level, with a total of 53 townships and districts being selected.

**Fig. 1:**
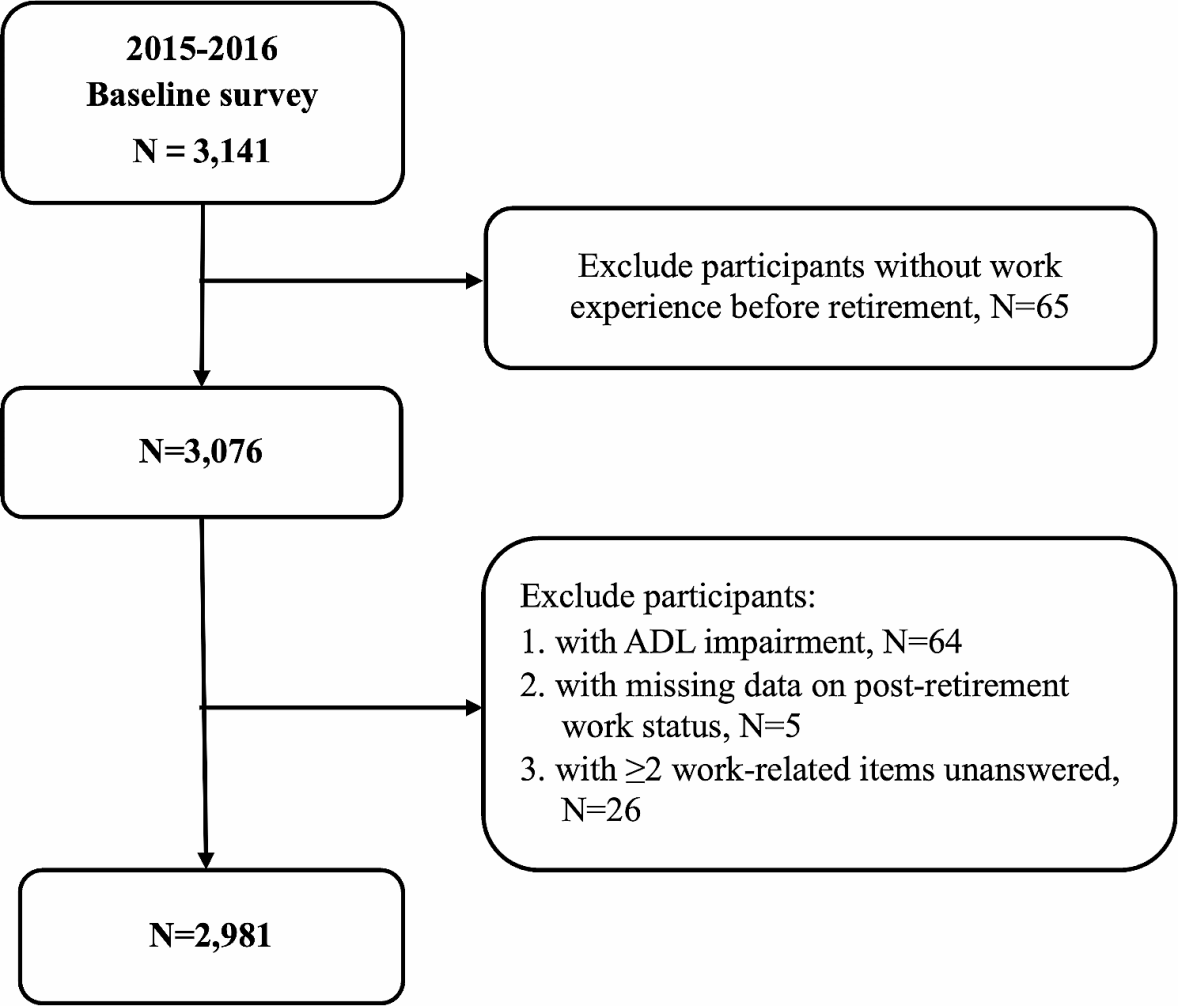
Participants exclusion diagram

The THRS was conducted from 2015 to 2016 through face-to-face interviews and ultimately had 1760 men (56%) and 1381 women (44%), which are a total of 3141 individuals as valid respondents who met the retirement criteria (pension recipients). This study analyzed factors associated with work status after retirement, including respondents’ sociodemographic information, self-rated health, economic status, and pre-retirement work-rated characteristics. The exclusion flowchart is shown in Fig. 1. The final number of participants included in the analysis was 2981.

### Measurement

#### Dependent variable: work status after retirement

This study defines retirement as the period following pension receipt and measures the work status after retirement by asking participants, “What is your current retirement status?“. Respondents were given seven response options: (1) Complete retirement with no part-time or work engagement; (2) Full-time wage employment with regular income; (3) Part-time wage employment with irregular income; (4) Unpaid full-time work (e.g., consultant); (5) Unpaid part-time work (e.g., chairman in an association, excluding volunteering); (6) Helping family businesses; and (7) Full-time grandchild care.

In response to research purposes, this study divided the 7 responses into four categories. Firstly, full-time and part-time paid employment with regular income were categorized as “paid work.” Meanwhile, helping family business was classified as “self-employment,” referring to paid work outside of paid employment [24]. Other responses, including full-time/part-time unpaid work and full-time grandparenting, were classified as “unpaid work.” The unpaid work did not include volunteering due to the limitation of the questions. Therefore, the classification of post-retirement work status in this study comprises four types: (1) Fully retired, (2) Paid work, (3) Self-employment, and (4) Unpaid work.

### Independent variables: work-related characteristics before retirement

Work-related characteristics included five factors: pre-retirement occupation, pre-retirement employment, pre-retirement work stress, pre-retirement job flexibility, and pre-retirement job autonomy.

### Pre-retirement occupation

Participants were asked, “What type of occupation did you have before retirement?“. Answers were categorized into three: (1) unskilled, (2) skilled/ semi-skilled, and (3) managerial/ professional.

### Pre-retirement employment

Participants were asked, “What was your employment before retirement?“. Answers were categorized into three: (1) government employee, (2) private sector employee, and (3) self-employed.

### Pre-retirement work stress

Participants were asked, “Did you feel stress at work?” and were given a 3-point Likert scale consisting of the following answers: (1) low, (2) medium, and (3) high.

### Pre-retirement work flexibility

Participants were asked, “Which statement best describes the scheduling of your primary job before retirement?” and were given three options: (1) low, (2) medium, and (3) high.

### Pre-retirement work autonomy

Participants were asked, “Which statement best describes the autonomy you had in your primary job before retirement?” and were given three options: (1) low, (2) medium, and (3) high.

### Other variables

Other variables included sociodemographic variables such as age (50–64, 65–74 years old), gender (male, female), education level (primary school or less, junior or high school, college or more), spouse/partner (yes, no), and years of retirement (0–5 years, ≥6years). Self-rated health (poor, fair, good) and economic difficulty (difficult, not difficult) were also measured herein.

### Statistical analysis

The study presents demographic characteristics and work-related factors in frequency and percentage stratified by gender (Table 1) and four types of post-retirement work status (Table 2). Spearman analysis was employed to examine the correlation between variables, and the Variance Inflation Factor (VIF) was used to clarify the issue of collinearity. Overall, the correlation between variables ranged from mild to moderate. In the collinearity test, the variable with the highest VIF was retirement pre-work time flexibility with a value of 2.12, which did not exceed the recommended value of 5, indicating that the collinearity of the variables included in the study is acceptable.

**Table 1:** Characteristics of participants in the study by gender

			Total (*n* = 2981)	Male (*n* = 1701)	Female (*n* = 1280)	
Variables	Categories		N (%)	N (%)	N (%)	P value
*Demographics factors*						
Age	50–64 years		1576 (52.9)	831 (48.9)	745 (58.2)	< 0.001
	65–74 years		1441 (47.1)	895 (51.2)	546 (41.8)	
Education	Primary school or less		962 (32.3)	455 (26.8)	507 (39.6)	< 0.001
	Junior or high school		1043 (35.0)	605 (35.6)	438 (34.2)	
	College or above		973 (32.7)	638 (37.6)	335 (26.2)	
Spouse/partner	No		524 (17.6)	175 (10.3)	349 (27.3)	< 0.001
	Yes		2452 (82.4)	1523 (89.7)	929 (72.7)	
Years after	0–5 years		1200 (40.8)	626 (37.2)	574 (45.5)	< 0.001
retirement	More than 6 years		1742 (59.2)	1055 (62.8)	687 (54.5)	
Self-rated health	Poor/not good		413 (13.9)	214 (12.6)	199 (15.6)	< 0.001
	Fair		1276 (42.8)	689 (40.5)	587 (45.9)	
	Excellent/good		1292 (43.3)	798 (46.9)	494 (38.6)	
Economic status	Not difficult/ Even		2146 (72.8)	1227 (72.8)	919 (72.9)	0.971
	Difficult		800 (27.2)	458 (27.2)	342 (27.1)	
*Work-related factors*						
Pre-retirement	Unskilled		594 (19.9)	193 (11.4)	401 (31.3)	< 0.001
occupations	Skilled/Semi-skilled		1645 (55.2)	1002 (58.9)	643 (50.2)	
	Managerial/Professional		742 (24.9)	506 (29.8)	236 (18.4)	
Pre-retirement	Government employee		935 (31.4)	605 (35.6)	330 (25.8)	< 0.001
employment	Private sectors		1349 (45.3)	704 (41.4)	645 (50.4)	
	Self-employment		696 (23.4)	392 (23.1)	304 (23.8)	
Pre-retirement	Low		1070 (35.9)	580 (34.1)	490 (38.3)	0.028
work stress	Medium		957 (32.1)	548 (32.2)	409 (32.0)	
	High		952 (32.0)	572 (33.7)	380 (29.7)	
Pre-retirement	Low		1888 (63.1)	1077 (63.4)	803 (62.7)	0.795
work flexibility	Moderate		513 (17.2)	295 (17.4)	218 (17.0)	
	High		586 (19.7)	327 (19.3)	259 (20.2)	
Pre-retirement	Low		1205 (40.4)	656 (38.6)	549 (42.9)	0.013
work autonomy	Moderate		981 (32.9)	595 (35.0)	386 (30.2)	
	High		794 (26.6)	449 (26.4)	345 (27.0)	
Work status	Fully retired		2197 (73.7)	1231 (72.4)	966 (75.5)	< 0.001
after retirement	Paid work		451 (15.1)	282 (16.6)	169 (13.2)	
	Self-employment		211 (7.1)	138 (8.1)	73 (5.7)	
	Unpaid work (merge)		122 (4.1)	50 (2.9)	72 (5.6)	
	-Unpaid work		58 (2.0)	31 (1.8)	27 (2.1)	
	-Grandparenting		64 (2.2)	19 (1.1)	45 (3.5)	

**Table 2:** Characteristics of participants by types of work status after retirement

		Fully Retired (*n* = 2197)	Paid work (*n* = 451)	Self-employment (*n* = 211)	Unpaid work (*n* = 122)	
Variables	Categories	N (%)	N (%)	N (%)	N (%)	P value
Gender	Male	1231 (72.4)	282 (16.6)	138 (8.1)	50 (2.9)	< 0.001
	Female	966 (75.5)	169 (13.2)	73 (5.7)	72 (5.6)	
Age	50–64 years	1072 (68.0)	318 (20.2)	108 (6.9)	78 (5.0)	< 0.001
	65–74 years	1125 (80.1)	133 (9.5)	103 (7.3)	44 (3.1)	
Education	Primary school or less	709 (73.7)	130 (13.5)	91 (9.5)	32 (3.3)	< 0.001
	Junior or high school	732 (70.2)	190 (18.2)	66 (6.3)	55 (5.3)	
	College or above	754 (77.5)	130 (13.4)	54 (5.6)	35 (3.6)	
Spouse/partner	No	374 (71.4)	100 (19.1)	30 (5.7)	20 (3.8)	0.031
	Yes	1819 (74.2)	350 (14.3)	181 (7.4)	102 (4.2)	
Years after	0–5 years	857 (71.4)	191 (15.9)	100 (8.3)	52 (4.3)	0.054
retirement	More than 6 years	1312 (75.3)	257 (14.8)	106 (6.1)	67 (3.9)	
Self-rated	Poor/not good	319 (77.2)	54 (13.1)	21 (5.1)	19 (4.6)	0.487
health	Fair	930 (72.9)	196 (15.4)	97 (7.6)	53 (4.2)	
	Excellent/good	948 (73.4)	201 (15.6)	93 (7.2)	50 (3.9)	
Economic	Not difficult/ Even	1612 (75.1)	310 (14.5)	144 (6.7)	80 (3.7)	0.049
status	Difficult	562 (70.3)	133 (16.6)	64 (8.0)	41 (5.1)	
Pre-retirement	Unskilled	439 (73.9)	95 (16.0)	33 (5.6)	27 (4.6)	< 0.001
occupations	Skilled/Semi-skilled	1183 (71.9)	257 (15.6)	139 (8.5)	66 (4.0)	
	Managerial/Professional	575 (77.5)	99 (13.3)	39 (5.3)	29 (3.9)	
Pre-retirement	Government employee	758 (81.1)	99 (10.6)	49 (5.2)	29 (3.1)	< 0.001
employment	Private sectors	989 (73.3)	241 (17.9)	59 (4.4)	60 (4.5)	
	Self-employment	450 (64.7)	111 (16.0)	103 (14.8)	32 (4.6)	
Pre-retirement	Low	1464 (77.9)	271 (14.4)	82 (4.4)	63 (3.4)	< 0.001
work stress	Medium	351 (68.4)	83 (16.2)	50 (9.8)	29 (5.7)	
	High	380 (64.9)	97 (16.6)	79 (13.5)	30 (5.1)	
Pre-retirement	Low	1464 (77.9)	271 (14.4)	82 (4.4)	63 (3.4)	< 0.001
work flexibility	Moderate	351 (68.4)	83 (16.2)	50 (9.8)	29 (5.7)	
	High	380 (64.9)	97 (16.6)	79 (13.5)	30 (5.1)	
Pre-retirement	Low	947 (78.6)	166 (13.8)	47 (3.9)	45 (3.7)	< 0.001
work autonomy	Moderate	691 (70.4)	164 (16.7)	75 (7.7)	51 (5.2)	
	High	558 (70.3)	121 (15.2)	89 (11.2)	26 (3.3)	

Multinomial logistic regression analysis was performed to explore the association between each independent variable and post-retirement work status. Complete retirement was used as the reference group while controlling for factors such as age, gender, years of retirement, educational level, marital status, perceived health status, and economic status. Additionally, to test the impact of gender differences, this study further conducted stratified analysis by gender to test the association of each variable with work status after retirement.

## Results

### Descriptions of participants

The demographic characteristics of the study sample are shown in Table 1. Except for the economic status, the distribution of participants across variables exhibited gender differences. According to the statistics on work-related factors, most participants belonged to the semi-skilled/skilled occupational type before retirement and were employed by private enterprises. Therefore, labor insurance was the most common way of receiving a pension. In addition to physical job demands and work flexibility before retirement, gender differences were also observed in the distribution of participants across various variables. Regarding the distribution of the dependent variable (post-retirement work status), most participants were fully retired (73.7%), while paid work, self-employment, and unpaid work accounted for 15.1%, 7.1%, and 4.1% of the remaining answers, respectively. The “unpaid work” comprised the most minor participants, less than 5%.

We then further conducted a bivariate analysis of retirement status and various variables. Results are shown in Table 2. For demographic factors, we found significant differences in retirement status distribution among gender, age, education level, marital status, and economic status. In terms of work-related factors, we also found significant differences in retirement status distribution among pre-retirement occupation, pre-retirement employment type, pre-retirement work stress, pre-retirement work flexibility, pre-retirement work autonomy, and type of pension.

### Factors associated with post-retirement work status

In Table 3, multinomial logistic regression analysis showed that work-related factors had different associations with post-retirement work statuses after controlling for covariates. Overall, the group that engaged in paid work after retirement tended to be male, younger (aged 50–64), had no spouse/partner, were previously employed in private enterprises or self-employed workers, and had moderate job autonomy before retirement. The group that engaged in self-employment after retirement tended to be male, with lower education, had lower job stress before retirement, higher job flexibility, and moderate job autonomy before retirement. Finally, the group that engaged in unpaid work after retirement tended to be female, younger, economically disadvantaged, and with higher job flexibility before retirement.

**Table 3:** Multinomial logistic regression for factors of work status after retirement (*n* = 2891)

Variables	Paid work OR (95%CI)		Self-employment OR (95%CI)	Unpaid work OR (95%CI)
*Work-related variables*				
1. Pre-retirement occupation (ref = unskilled staff)				
Skilled/Semi-skilled staff	0.86 (0.64–1.16)		1.13 (0.73–1.75)	0.96 (0.57–1.62)
Managerial/Professional	0.90 (0.59–1.37)		0.87 (0.46–1.64)	1.28 (0.61–2.67)
2. Pre-retirement employment (ref = Government employee)				
Private sectors	1.95 (1.44–2.65)		0.76 (0.48–1.21)	1.58 (0.91–2.75)
Self-employment	1.81 (1.16–2.82)		1.64 (0.91–2.93)	1.40 (0.66–2.94)
3. Pre-retirement work stress (ref = high)				
Low	1.01 (0.78–1.32)		1.78 (1.18–2.67)	0.92 (0.59–1.43)
Medium	0.81 (0.62–1.06)		1.85 (1.23–2.76)	0.51 (0.31–0.84)
4. Pre-retirement work flexibility (ref = low)				
Moderate	1.10 (0.79–1.52)		1.61 (1.00-2.57)	1.86 (1.08–3.19)
High	1.41 (0.93–2.13)		1.96 (1.13–3.40)	3.17 (1.63–6.20)
5. Pre-retirement work autonomy (ref = low)				
Moderate	1.45 (1.09–1.92)		1.73 (1.10–2.72)	1.26 (0.77–2.07)
High	0.96 (0.66–1.39)		1.33 (0.77–2.28)	0.43 (0.22–0.87)
*Other variables*				
6. Gender (ref = female)				
Male	1.71 (1.35–2.16)		1.63 (1.17–2.28)	0.55 (0.37–0.83)
7. Age (ref = 65–74 years)				
50–64 years	3.00 (2.34–3.85)		1.25 (0.90–1.73)	1.70 (1.11–2.62)
8. Education (ref = primary school and less)				
Junior and senior high school	1.17 (0.89–1.54)		0.65 (0.45–0.94)	1.75 (1.06–2.90)
College and more	0.92 (0.63–1.33)		0.66 (0.40–1.10)	1.26 (0.63–2.51)
9. Spouse/partner (ref = yes)				
No	1.69 (1.29–2.22)		0.86 (0.55–1.33)	0.75 (0.43–1.29)
10. Years after retirement (ref = ≥ 6 years)				
0–5 years	0.76 (0.60–0.95)		1.19 (0.86–1.64)	0.83 (0.55–1.25)
11. Self-rated health (ref = poor/not good)				
Fair	1.26 (0.89–1.79)		1.66 (0.99–2.79)	0.99 (0.56–1.76)
Excellent/good	1.34 (0.94–1.91)		1.67 (0.98–2.83)	0.96 (0.53–1.73)
12. Economic status (ref = not difficult/even)				
Difficult	1.11 (0.87–1.42)		1.20 (0.85–1.69)	1.52(1.00-2.31)

### Gender differences in post-retirement work status

In the analysis of multinomial logistic regression stratified by gender, Table 4 showed that for men, after controlling for various variables, pre-retirement work stress has an effect on the choice of self-employment after retirement. Compared to those with high work stress, the likelihood of choosing self-employment after retirement is higher for those with medium or low work stress, with odds ratios of 2.30 (95% CI: 1.40–3.76) and 1.76 (95% CI: 1.05–2.94), respectively. However, there was no statistically significant difference for women: when women had medium work stress before retirement, the odds of participating in unpaid work after retirement were lower than those with high work stress, with an odds ratio of 0.30 (95% CI: 0.15–0.62).

**Table 4:** Multinomial logistic regression for factors of work status after retirement by gender

		Male			Female	
Variables	Paid work OR (95%CI)	Self-employment OR (95%CI)	Unpaid work OR (95%CI)	Paid work OR (95%CI)	Self-employment OR (95%CI)	Unpaid work OR (95%CI)
*Work-related variables*						
1. Pre-retirement occupation (ref = unskilled staff)						
Skilled/Semi-skilled staff	0.87 (0.56–1.38)	0.70 (0.38–1.29)	0.75 (0.29–1.96)	0.91 (0.61–1.37)	1.80 (0.95–3.41)	1.15 (0.62–2.13)
Managerial/Professional	0.94 (0.54–1.64)	0.61 (0.28–1.35)	0.83 (0.26–2.60)	0.96 (0.44–2.11)	1.16 (0.34–3.92)	2.01 (0.70–5.77)
2. Pre-retirement employment (ref = government employee)						
Private sectors	1.79 (1.25–2.56)	0.69 (0.40–1.21)	1.31 (0.62–2.76)	2.52 (1.34–4.73)	0.84 (0.32–2.22)	2.30 (0.97–5.48)
Self-employment	1.76 (0.99–3.10)	2.33 (1.13–4.82)	0.71 (0.22–2.30)	2.30 (1.05–5.06)	1.16 (0.39–3.46)	2.94 (1.00-8.65)
3. Pre-retirement work stress (ref = high)						
Low	1.03 (0.74–1.44)	1.76 (1.05–2.94)	1.24 (0.61–2.53)	0.96 (0.62–1.48)	1.49 (0.76–2.94)	0.71 (0.40–1.29)
Medium	0.89 (0.63–1.24)	2.30 (1.40–3.76)	0.93 (0.45–1.92)	0.69 (0.44–1.08)	1.05 (0.51–2.16)	0.30 (0.15–0.62)
4. Pre-retirement work flexibility (ref = low)						
Moderate	1.14 (0.76–1.72)	1.09 (0.60–1.97)	3.38 (1.60–7.13)	0.91 (0.52–1.60)	3.34 (1.42–7.82)	0.95 (0.42–2.17)
High	1.15 (0.67–1.99)	1.33 (0.65–2.72)	4.21 (1.45–12.21)	1.77 (0.93–3.36)	3.73 (1.45–9.57)	2.50 (1.02–6.16)
5. Pre-retirement work autonomy (ref = low)						
Moderate	1.21 (0.85–1.72)	1.95 (1.14–3.35)	1.09 (0.52–2.28)	1.94 (1.20–3.14)	1.13 (0.47–2.71)	1.53 (0.77–3.04)
High	0.98 (0.61–1.56)	1.23 (0.62–2.43)	0.37 (0.12–1.12)	0.90 (0.48–1.67)	1.33 (0.52–3.42)	0.48 (0.19–1.21)
*Other variables*						
6. Age (ref = 65–74 years)						
50–64 years	3.05 (2.25–4.14)	1.36 (0.91–2.04)	1.90 (0.99–3.64)	2.91 (1.89–4.48)	1.18 (0.65–2.13)	1.58 (0.88–2.85)
7. Education (ref = primary school and less)						
Junior and senior high school	1.21 (0.83–1.74)	0.70 (0.43–1.13)	2.42 (0.97–6.07)	1.23 (0.81–1.88)	0.56 (0.30–1.04)	1.54 (0.82–2.88)
College and more	1.00 (0.64–1.59)	0.80 (0.43–1.49)	2.03 (0.67–6.13)	0.74 (0.38–1.46)	0.43 (0.16–1.15)	0.81 (0.30–2.18)
8. Spouse/partner (ref = yes)						
No	1.46 (0.97–2.19)	0.90 (0.47–1.71)	0.18 (0.03–1.33)	1.88 (1.29–2.75)	0.73 (0.38–1.37)	0.92 (0.50–1.70)
9. Years after retirement (ref = ≥ 6 years)						
0–5 years	0.68 (0.51–0.92)	1.14 (0.76–1.70)	0.77 (0.40–1.47)	0.91 (0.63–1.32)	1.26 (0.72–2.21)	0.86 (0.50–1.47)
10. Self-rated health (ref = poor/not good)						
Fair	1.46 (0.91–2.36)	1.78 (0.91–3.46)	0.53 (0.23–1.22)	1.01 (0.60–1.68)	1.43 (0.61–3.36)	1.54 (0.68–3.52)
Excellent/good	1.53 (0.95–2.45)	1.31 (0.66–2.58)	0.52 (0.23–1.18)	1.10 (0.64–1.91)	2.63 (1.11–6.23)	1.66 (0.70–3.95)
11. Economic status (ref = not difficult/even)						
Difficult	0.97 (0.71–1.33)	1.02 (0.66–1.57)	1.47 (0.77–2.82)	1.40 (0.95–2.06)	1.57 (0.88–2.80)	1.63 (0.93–2.86)

The impact of work flexibility before retirement on post-retirement work status is contrasted between men and women. For the former, those with moderate or high work flexibility before retirement were more likely to choose unpaid work after retirement compared to those with low work flexibility, with odds ratios of 3.38 (95% CI: 1.60–7.13) and 4.21 (95% CI: 1.45–12.21), respectively. For the latter, the work flexibility before retirement also had an impact on choosing self-employment and unpaid work after retirement. Those with moderate or high work flexibility were more likely to choose self-employment after retirement, with odds ratios of 3.34 (95% CI: 1.42–7.82) and 3.73 (95% CI: 1.45–9.57), respectively. Those who chose unpaid work tended to have high work flexibility, with an odds ratio of 2.50 (95% CI: 1.02–6.16) compared to those with low work flexibility.

For work autonomy before retirement, the impact on post-retirement work choices contrasted between men and women. Men with moderate work autonomy before retirement were more likely to choose self-employment after retirement, in contrast to women with moderate work autonomy before retirement, who were instead more likely to choose paid work after retirement.

## Discussion

This study mainly explores the factors influencing retirees’ choice of working status after retirement. In addition to paid jobs such as employed or self-employed work, the study also investigated the possible influencing factors of unpaid work. The results of the analysis provide a profound understanding of the decision-making of retired individuals to continue working as references for government policy. Results show that those who engage in paid work after retirement tend to be male, younger, unmarried or without a partner, employed in a private enterprise or self-employed before retirement, and had moderate pre-retirement work autonomy. Meanwhile, those who engage in self-employed work after retirement tend to be male, with lower educational levels, lower pre-retirement work pressure, more flexible work hours, and moderate work autonomy. Finally, those who engage in unpaid work after retirement tend to be female, younger, with a high school education, in challenging economic conditions, and with higher pre-retirement work flexibility and lower work autonomy.

### Work-related characteristics before retirement

For work-related factors before retirement, this study investigates the influence of pre-retirement work stress, pre-retirement work flexibility, and pre-retirement work autonomy on engaging in different types of work after retirement. We found that pre-retirement work stress indeed affects the work decisions of older workers after retirement. Compared to those with high pre-retirement work stress, those with moderate- or low-stress levels are more likely to engage in self-employment after retirement. This is similar to von Bonsdorff, Zhan, Song, and Wang’s [25] view, which suggests that self-employment may be one way to exit from unpleasant work. This study’s finding emphasizes the importance of the psychological well-being of older workers before retirement; thus, providing appropriate psychological interventions towards the end of their careers may help prolong their work lives.

The present study also discovered that pre-retirement work flexibility contributes to extending the work life of middle-aged and older individuals. Hudomiet, Hurd, Parker, and Rohwedder [26] identified that options for flexible work hours in the workplace effectively increase the willingness of older adults to continue working post-retirement. Analyzing data from a representative sample in Taiwan, Lu, Kao, Chang, Wu, and Cooper [27] also found that pre-retirement flexibility in work hours contributes to reducing work-family interference, further enhancing job satisfaction and organizational commitment, both of which are positive factors for continued work post-retirement. On the other hand, Rhee, Park, and Lee [28] suggested that workplace flexibility indirectly reduces workers’ intentions to leave by reducing work-family conflicts and boosting job satisfaction. This study further found that retired workers with higher levels of prior work flexibility also benefited from continuing work, particularly for those engaged in self-employment, showing significant differences. These results offer valuable policy insights, suggesting that providing reasonable work flexibility for middle-aged and older workers could be a friendly strategy for extending work life. However, this survey did not know whether participants continued working in their previous workplaces or became self-employed, and those with higher work flexibility were potentially more inclined toward self-employment; thus, the causality between the two is warranted for future study.

For the variable of job autonomy before retirement, results found that compared to those with low job autonomy, individuals with moderate job autonomy had a higher ratio of engaging in paid and self-employment work after retirement. Previous studies have shown that job autonomy is another critical factor for successful employment transition among older employees [29], mainly because it can shape a proactive work environment for older workers, increasing their motivation to continue working in the workforce. Hansson, Buratti, Johansson, and Berg [30] found that although poor health status and economic conditions can affect the life satisfaction of retirees, the adverse effects can be compensated for by increasing job autonomy. However, job autonomy is not necessarily beneficial for continued work after retirement, especially when it is too high. Highly autonomous workers may not necessarily have a higher ratio of engaging in paid or self-employment work. They may even significantly decrease the ratio of engaging in unpaid work. Current research on the relationship between job autonomy and continued work after retirement remains very limited, and the underlying mechanisms remain unclear. More succeeding research is thus needed to clarify the causal relationships and effects among these factors.

This study also found that individuals in pre-retirement occupations, such as those in private or self-employed sectors before retirement, were more likely to engage in paid work after retirement than those who worked in government before retirement. This may be related to the retirement system in Taiwan, where public servants are restricted from returning to public service due to retirement benefits regulations, which hinder their engagement in paid work after retirement [31]. Private enterprises in Taiwan also often require employees to claim retirement benefits upon reaching the statutory retirement age due to considerations regarding retirement fund management, followed by rehiring those employees later. Notably, this study found no significant correlation between educational level and post-retirement work: Individuals with vocational or university degrees were even less likely to continue working after retirement. However, previous studies have often considered education level and pre-retirement occupation as essential factors for post-retirement work, and individuals with higher education are usually more likely to continue working after retirement [32, 33]. Future research is thus needed to clarify the effect of educational level on post-retirement work, which may be influenced by the retirement system or cultural context of other countries.

### Other variables

#### Age

The results of this study are consistent with existing research on the association between age and continuing work after retirement. Most studies have supported age as an important factor in continuing work after retirement, with younger individuals having a higher tendency to choose to continue working [20, 22, 34–39].. This inclination might stem from older employees’ challenges in meeting job demands due to the declines in physiological and cognitive functions [37] and could also affect work motivation [23]. This study further found that the association between age and the choice of continuing work after retirement is somewhat different. Age is only significantly associated with paid and unpaid work but not self-employment, with a higher chance of engaging in paid work. The possibility of younger individuals engaging in paid work after retirement is also three times higher than that of older individuals, but there is no statistically significant difference in those who engage in self-employed work after retirement. This could be related to the sustainability of self-employment [40]. Coincidentally, von Bonsdorff, Zhan, Song, and Wang [25] believe that self-employment can serve as a transitional job for middle-aged and elderly individuals before full retirement and can sustainably enter the labor market longer than salaried workers.

#### Self-rated health

For self-rated health, this study did not find a statistically significant correlation with post-retirement work in both bivariate and multivariate analyses. However, it did find that women who rated their health as “good” were 2.56 times more likely to engage in self-employment after retirement than those who rated their health as “poor.” Many previous studies have supported health status as an important predictor of post-retirement work [41–43]. However, this study did not find a significant correlation between self-rated health and post-retirement work. There may be sample selection bias due to the healthy worker effect, as the study excluded those with functional impairments in daily living. Therefore, the participants may be relatively healthier than the general population. Furthermore, self-rated health is based on subjective judgments of one’s health status and is therefore not objective.

### Economic status

Another variable worth exploring is economic status. Previous studies have suggested that financial situation is essential for older workers to continue working after retirement [12, 23, 24, 36, 39, 44]. However, the results of our analysis do not support this, and Beutell and Schneer [20] also found that economic factors may not be the core factors in choosing bridge employment. Although the situation where older workers stay in the labor market for financial reasons allows them to meet their practical needs, it is necessary to consider personal retirement preparation and social security welfare comprehensively. For example, in the United States, medical insurance can incentivize older workers to continue working after retirement [20, 45]. However, such incentives could not be applicable in Taiwan, where institutional or cultural factors such as inadequate post-retirement labor insurance support, age discrimination, and family values, as mentioned in Huang’s doctoral thesis [46], may lead to older workers being unable to continue working. This means Taiwan’s elderly population is often unwilling to participate in the labor market or social activities [47].

This study also found another interesting result: economic status is related to unpaid work after retirement. Those who perceive their financial situation as more difficult are 1.52 times more likely to engage in unpaid work after retirement than those with normal or no difficulties. Especially women mainly took on family care responsibilities, such as caring for grandchildren or engaging in caregiving work. Unpaid work may therefore still have a specific labor value. For example, taking care of grandchildren can reduce the cost of hiring a babysitter or compensate for wage losses due to caregiving needs. This ultimately becomes an important substitute workforce for families with economic burdens.

### Gender differences

The study also revealed distinct gender differences in post-retirement employment. Men tended to engage in paid work and self-employment, while women were inclined toward unpaid work. Gender’s impact on the likelihood of continued work after retirement remains inconclusive. Some studies suggest a higher probability of working among women, as they might re-enter the workforce to compensate for economic losses due to caregiving responsibilities [48]. Other researchers suggest that men are more likely to continue working after retirement [33], especially in Asian countries where men traditionally hold the primary role in providing financial support and are less likely to retire fully [31].

This study also found significant gender differences in post-retirement employment. Men tend to engage in paid and self-employed work, while women tend to engage in unpaid work, which may be related to cultural contexts in Taiwan. Traditional Taiwanese beliefs still regard men as the primary breadwinners, while women are considered to be responsible for caring for the family. For instance, Beutell and Schneer [20] found that women are more inclined to leave the job market to care for their families or grandchildren.

Retirement-related factors (pre-retirement job stress and job autonomy) also exhibit gender differences. Pre-retirement job stress is a driving factor for self-employment among male retirees but is also an influencing factor for unpaid work among female retirees. Moreover, moderate pre-retirement job autonomy is attractive for self-employment among male retirees but tends to lead female retirees towards paid work. Future research is suggested to explore the types of jobs and trajectories after retirement, which may allow for further understanding of these underlying mechanisms. However, this study is limited by cross-sectional data and cannot reveal the trajectory of changes over time. Therefore, large national representative databases are expected to incorporate job-related factors to clarify the underlying mechanisms.

## Limitations

Several potential limitations in this study must be addressed. Firstly, although this study collected various retirement-related factors from the retired population, it is essentially a cross-sectional study and can thus only conduct exploratory correlation tests, not causal inferences. Secondly, the data collection tool used herein was a self-report questionnaire, which is more subjective and prone to recall bias. Thirdly, although the items related to work factors were taken from a developed questionnaire, this study only extracted one item and lacked related reliability and validity tests. Therefore, we suggest that future studies use a fully developed questionnaire to re-examine the issue. Fourthly, this study did not have information on participants’ family savings, personal and spousal pension, or details regarding the spouse’s employment status and health condition. Fifthly, there was a lack of pre-retirement organizational work context-related factors. Past studies have found that this information affects their post-retirement work, but only a few databases are available for obtaining relevant information. It is recommended that similar long-term tracking databases be constructed to compensate for the current knowledge gap. Lastly and most importantly, this study defines retirement as receiving a retirement pension. However, this does not imply that individuals have completely exited the labor market. Nevertheless, the findings of this study contribute to clarifying the impact of work-related characteristics on retirement decisions. This study advocates for and looks forward to future research continuing to uncover new evidence and explore influencing mechanisms.

## Conclusion

This study reveals that sociodemographic factors such as gender, age, marital status, and economic status are associated with post-retirement work continuation. Work-related factors before retirement, such as employment type, job stress, work flexibility, and job autonomy, also impact individuals’ work after retirement. Understanding the preferences of older workers regarding job types, availability, and how job conditions influence retirement decisions is crucial. Unlike factors such as age, marital status, and education level, which are irreversible or challenging to change, pre-retirement work characteristics offer effective strategies for promoting employment. Thus, implementing reasonable accommodations, such as flexible work hours, intergenerational collaboration, job sharing, or phased retirement schemes, can contribute to continued labor participation among older adults, embodying the concept of active aging. This approach can support policy development, offering complementary measures for postponing mandatory retirement ages.

Furthermore, this study underscores the substantial impact of gender roles on post-retirement work choices, emphasizing the need for gender-specific measures in policymaking to promote employment among older people. Government research into retirement systems and guidance or incentive measures for different sexes is recommended.

## Data Availability

Data can be shared upon request to the corresponding author, with permission from the Health Promotion Administration, Ministry of Health and Welfare, Taiwan.
